# Concealed penis in pediatric age group: a comparison between three surgical techniques

**DOI:** 10.1186/s12894-022-01169-3

**Published:** 2023-01-11

**Authors:** Ahmed Elrouby

**Affiliations:** Faculty of Medicine, Alexandria University, Alexandria, Egypt

**Keywords:** Concealed, Phallopexy, Dartos excision, Degloving, Penile re-retraction

## Abstract

**Background:**

Comparison between three different surgical techniques in the management of concealed penis.

**Methods:**

This prospective interventional non-randomized study included 150 pediatric patients with a concealed penis. They were distributed equally into three groups; group A; patients treated by anchoring the penile skin dermis to Buck's fascia at the penile base at 3 and 9 o'clock points using PDS 5/0 (phallopexy), group B; patients treated by complete dissection and excision of dartos fascia and group C; patients treated by phallopexy as in group A after complete dissection and excision of dartos fascia. Follow-up at the end of the 1st post-operative week and then monthly for 6 months as regards penile skin congestion and/or necrosis, wound infection, edema, and/or re-retraction was carried out.

**Results:**

Penile edema and re-retraction have a statistically significant difference among the studied groups (p < 0.001 and p = 0.002 respectively). Penile re-retraction was noticed to be lowest in patients of group C, however penile edema was observed to be highest in patients of group B.

**Conclusions:**

Phallopexy after complete dissection and excision of dartos fascia have better results than doing either phallopexy or dartos excision alone in the treatment of concealed penis.

*Clinical trial registration*: The manuscript was registered in ClinicalTrials.gov Protocol Registration and Results System. ClinicalTrials.gov Identifier: NCT05565040. Our manuscript was registered on 4/10/2022.

## Background

**Fig. 1:**
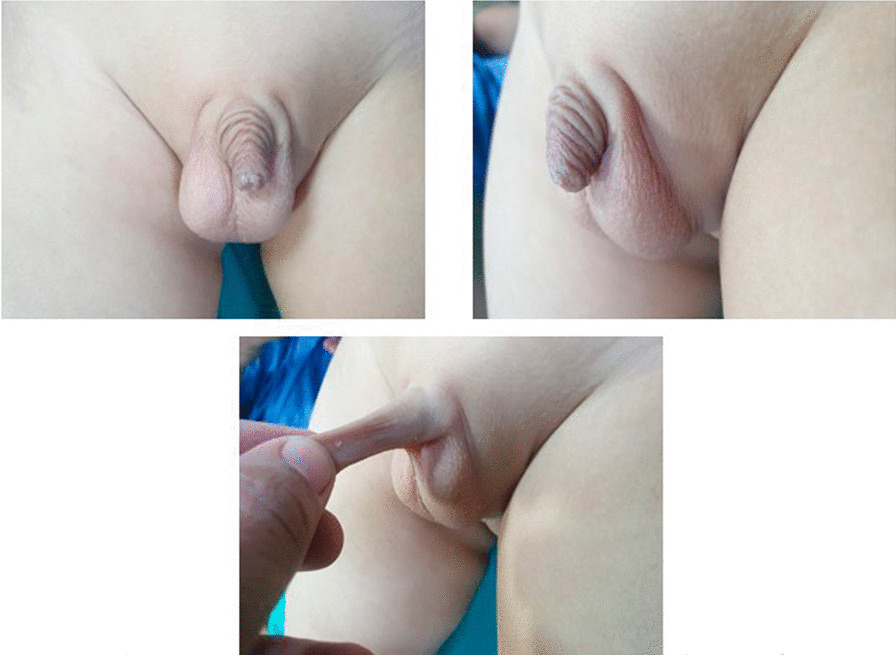
Concealed penis

The concealed penis is a congenital anomaly in which a normal size penis is hidden in the pre-pubic fat with the glans penis that does not project from the pubic or scrotal skin (Fig. 1). This condition is usually associated with poor cosmoses, difficult accessibility resulting in poor hygiene, social embarrassment, recurrent balanitis, difficult urination, and secondary phimosis [1, 2].

The pathogenesis of this anomaly was attributed to an excessive development of the penile dartos fascia retracting the penis inwards, an insufficient attachment of the penile skin to the deep penile tissues at the penile base, and/or tight phimosis which is often present. Also, excessive pre-pubic fat is usually present and worsens the appearance of the abnormality but does not explain the pathology by itself [3].

Another variety of buried penis is the congenital mega-prepuce in which a redundant inner preputial skin covers the glans penis in an average size penis in association with severe phimosis. These patients usually complain of voiding difficulties as the urine traps in the redundant dome-like mega-prepuce. This variety requires different treatment modalities [4].

The difference in the outcome of different corrective techniques for correction of the concealed penis was not compared widely in the literature. So, the aim of this study is to compare the outcome of three different surgical techniques used in the management of such conditions in the pediatric age group. The 1st technique is in the form of anchoring the penile skin dermis to Buck's fascia (phallopexy) at the penile base, the 2nd one is in the form of complete dissection and excision of dartos fascia and the 3rd technique is in the form of phallopexy in association with complete dissection and excision of dartos fascia.

## Methods

This study is a prospective interventional non-randomized study. It included 150 uncircumcised patients in the pediatric age group who presented to Elshatby University Hospital with a concealed penis between June 2018 and June 2021. The main complaint of the parents of the studied patients was appearing small size penis, unapparent penis, difficult cleaning, and/or un-satisfaction.

The inclusion criteria included patients with a buried penis at or below the pubic skin level with a stretched penile length for all of them within the normal range according to their ages as titrated in the literature [5].

Patients with any associated anomalies like hypospadias, torsion, penoscrotal web, micropenis, mega-prepuce, or chordae were excluded from the study.

The included patients were distributed non-randomly into three groups. The procedure was chosen according to the operative findings as dartos dissection and excision were recommended in patients with a thick abnormal dartos alone, phallopexy was recommended in case of absence of normal penile fixation alone and a combination of both techniques was recommended when the two pathologies were found. Group A included patients who were treated by anchoring the penile skin dermis to Buck's fascia (phallopexy) at the penile base without excision of dartos fascia, group B included patients who were treated by complete dissection and excision of dartos fascia only and group C included patients who were treated by phallopexy as in patients of group A but after complete dissection and excision of dartos fascia.

All of the studied patients had routine pre-operative laboratory investigations including CBC, CT, BT, PT, PTT, and INR. Informed consent was signed by the parents or caregivers after a complete explanation of the study design, its publication as well as any possible complications.

All of the studied patients were operated on under general anesthesia with skin preparation using povidone-iodine. The prepuce was also retracted backward and cleaned completely from any smegma with povidone-iodine.

**Fig. 2:**
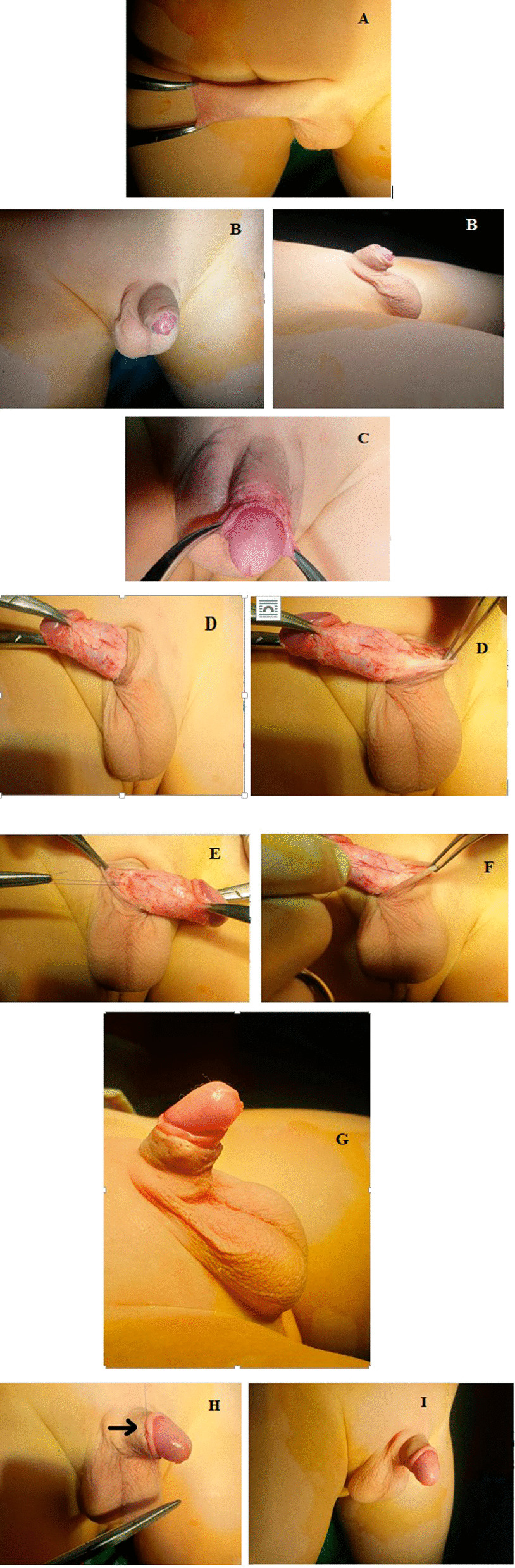
The steps of the procedure. **A** The beginning of the procedure by formal circumcision; two mosquitos pulling the mucocutaneous junction at 6 and 12 o’clock. **B** Immediately after circumcision with the penis retracted inside the penile skin. **C** The start of complete penile degloving with two mosquitos applied at 3 and 9 o’clock pulling the penis outward to facilitate dissection. **D** The end of penile degloving with complete excision of the abnormal tethering dartos fascia. **E**, **F** Phallopexy; fixation of penile Buck’s fascia to the penile dermis at the penile base at 3 o’clock and 9 o’clock respectively. **G** The totally corrected concealed penis at the end of the procedure (Before muco-cutaneous closure). **H** Subcuticular closure of muco-cutaneous junction. **I** The end of the procedure after closure of muco-cutaneous junction

The procedure was started by the application of two forceps mosquitos at the preputial mucocutaneous junction with formal circumcision leaving about 5 mm of the mucosal collar from the sub-coronal level (Fig. 2A). An inward penile retraction inside the penile skin was noticed in all patients immediately after circumcision as shown in Fig. 2B. This is followed by the application of two curved mosquito forceps at the sub-coronal level at three and nine o'clock points retracting the penis outwards during the whole procedure taking care of non-rotating the penis along with the operation by keeping retraction in the mid-line continuously (Fig. 2C).

Complete penile degloving in the sub-dartos plane was then started with meticulous dissection of the skin and dartos fascia out of Buck's fascia down to the level of penoscrotal junction ventrally and penopubic angle dorsally taking care of both neurovascular bundle and skin vascularity. On the dorsal aspect; the fundiform ligament—which is usually attached to the distal or mid-penile shaft—was identified and dissected to increase the penile length. The dissection was completed circumferentially releasing all of the abnormal bands anchoring the dartos fascia to Buck's fascia (Fig. 2D).

To avoid penile re-retraction; one of the following techniques was done (Fig. 2B). In patients of the group, a phallopexy was done in the form of anchoring the penile Buck's fascia to the penile skin dermis at the penile base at three and nine o'clock points without excision of dartos fascia. This was done using PDS 5/0 taking care of non-rotating the penis while taking the stitches by keeping retraction just in the midline. Patients of group B were managed by complete circumferential dissection and excision of dartos fascia only. This was done in a meticulous fashion taking care of skin vascularity. Lastly, patients of group C were managed by phallopexy as in patients of group A but after complete dissection and excision of dartos fascia (Fig. 2E, F).

After removal of the retracting forceps; the skin was repositioned over the degloved penis and was sutured distally to the mucosal collar in a sub-cuticular fashion using Vicryl 6/0 (Fig. 2G–I). A compression dressing was applied over the wound after the application of local antibiotic cream.

Removal of the dressing was done on the 1st postoperative day with repeated application of local antibiotic cream three times daily for 1 week in addition to oral anti-inflammatory (NSAIDs) and anti-edematous for 5 days.

The follow-up was carried out at the outpatient clinic by the resident of pediatric surgery who was blinded by the surgical technique. Follow-up of the appearance of the penile skin (congestion, necrosis), wound infection, edema, and or re-retraction at the end of the 1st post-operative week and then monthly for 6 months was done. Also. Comparing the follow-up results between the three studied groups was carried out using the appropriate statistical method.

### Statistical analysis

Data were coded, reviewed, and analyzed using the SPSS version 25.0 (Armonk, NY: IBM Corp). The Kolmogorov–Smirnov (KS) test was used to test the normal distribution of the data. Quantitative data were expressed as median and range. Qualitative data were expressed as frequency and percentage. Kruskal–Wallis test was used to detect any statistically significant differences between three or more independent non-normally distributed groups. A pairwise comparison was conducted among significant groups. The Chi-square test was used to test the association between two categorical variables or to detect the difference between two or more proportions and whenever χ2 was not valid, Monte Carlo exact probability was used for RxC tables. The statistical analysis was carried out in the Community Department, Faculty of Medicine, Alexandria University.

### Results

The mean age of the studied patients was 19.42 ± 20.93 months, while the mean weight was 11.97 ± 5.80 kg (Table 1).

**Table 1:** Description of the age and weight of the studied patients

Variables	Mean ± SD	Median
Age (Months)	19.42 ± 20.93	12.5 (4–144)
Weight (kg)	11.97 ± 5.80	11.3 (6–48)

However as the data were non-parametric, the median was used for its expression as well as for comparison of the studied groups. The median of the patient’s age and weight was significantly different among the three studied groups; being highest in group B and lowest in group A as shown in Table 2 (p-value for age = 0.004 and p-value for weight = 0.001).

**Table 2:** Comparison between the three studied groups as regards age and weight

Variables	Surgical techniques			Test of significance-value)
	Group A (n = 50)	Group B (n = 50)	Group C (n = 50)	
*Age (Months)*				H = 10.943 p = 0.004*
Mean ± SD	17.0 ± 23.76	20.84 ± 16.62	20.02 ± 21.49	
Median	8 (4–129)	15 (5–108)	13 (4–144)	
Significance between the studied groups	p1 = 0.005*, p2 = 0.039*, p3 = 1.0			
*Weight (kg)*				H = 13.460 p = 0.001*
Mean ± SD	11.02 ± 5.8	13.31 ± 4.79	11.59 ± 6.54	
Median	8.5 (6.5–40)	12 0(7–36)	9 (6–48)	
Significance between the studied groups	p1 = 0.001*, p2 = 0.87, p3 = 0.036*			

H; Kruskal Wallis test, Pairwise comparison was conducted using Bonferroni test

p; p-value between the three surgical groups

p1; p-valuee between group A and B

p2; p-valuee between group A and C

p3; p-valuee between group B and C

*Significant (p < 0.05)

The operative time was almost the same in the three studied groups being about 35 min without significant difference among the three studied groups.

The follow-up of the studied patients at the end of the 1st post-operative week and then monthly for 6 months as regards the appearance of the penile skin (congestion, necrosis), wound infection, penile edema, and/or re-retraction revealed different incidences among the studied groups with variable significance as shown in Table 3

**Table 3:** Comparison between the three studied groups as regards the follow-up results

Variables	Surgical techniques						Test of significance p-e)
	Group A (n = 50)		Group B (n = 50)		Group C (n = 50)		
	No	%	No	%	No	%	
*Skin congestion*							^MC^p = 0.122
Absent	49	98.0	45	90.0	49	98.0	
Present	1	2.0	5	10.0	1	2.0	
*Skin infection*							^MC^p = 1.0
Present	0	0.0	1	2.0	0	0.0	
Absent	50	100.0	49	98.0	50	100.0	
Penile edema							X^2^ = 34.720 p < 0.001*
Yes	35_a_	70.0	39_a_	78.0	38_b_	76.0	
No	15_a_	30.0	11_a_	22.0	12_b_	24.0	
*Penile re-retraction*							X^2^ = 12.444 p = 0.002*
Yes	3_a_	6.0	11_b_	22.0	1_a_	2.0	
No	47_a_	94.0	39_b_	78.0	49_a_	98.0	

^MC^p, Monte Carlo exact probability; X^2^, Chi square test

*Significant (p < 0.05)

^a,b^Different letters indicate a significant difference between column proportion

None of the studied patients have post-operative penile skin necrosis however, skin congestion was noticed in seven patients (4.7%) with gradual spontaneous improvement. The difference in the incidence of penile skin congestion among the studied groups was not statistically significant (Table 3, p = 0.103).

Only one patient (0.7%) developed a minor post-operative skin infection; this patient belonged to group B; this difference was not statistically significant (Table 3, p = 1.0). This patient was managed by maintaining treatment for ten postoperative days showing gradual improvement.

Most of the studied patients (n = 112, 74.7%) developed post-operative penile skin edema with different incidences among the studied groups. This difference was significantly different with the lowest rate in group A and the highest in group B as shown in Table 3 (p < 0.001*).

**Fig. 3:**
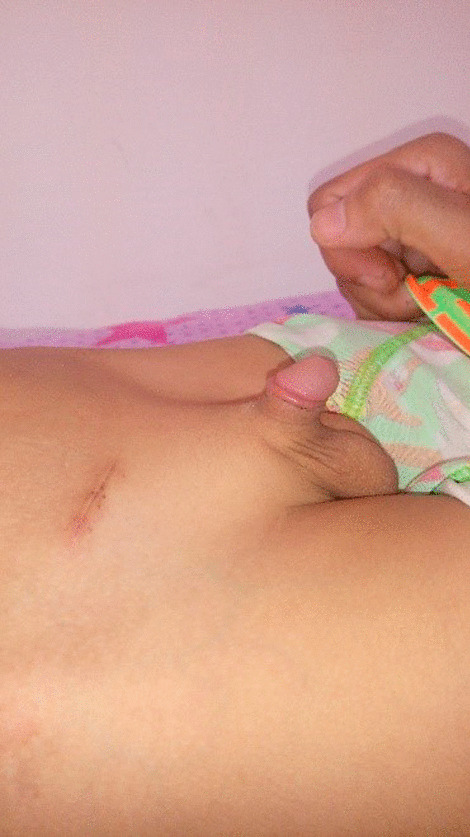
Post-operative appearance of a patient treated by a combination of phallopexy and dartos excision

Fifteen of the studied patients (10%) developed post-operative penile re-retraction with redundant skin; from whom the highest incidence was noticed in group B (n = 11, 73%) followed by group A (n = 3, 20%) while the lowest incidence was detected in group C (n = 1, 7%). This difference was statistically significant as shown in Table 3 (p = 0.002*) (Fig. 3).

The effect of age and weight at the operation of the studied patients on the postoperative follow-up outcome was tested and showed no statistical significance except for penile re-retraction (p = 0.006 and 0.003 for age and weight, respectively). Also, there was no significant correlation between the operative time and surgical outcome.

## Discussion

Several techniques had been described for the management of concealed penis without a single procedure showing complete satisfactory results. These procedures include; phallopexy alone (anchoring of penile skin dermis to Buck's fascia at penile base), dartos excision alone, and phallopexy after complete dissection and excision of dartos fascia. Excision of dartos fascia -which was done in patients of Group B as an only step and in patients of group C in combination with phallopexy in our study- was recommended for surgical correction of such conditions by several researchers like Alter et al. [6] who described in their study that true concealed penis results mainly from the presence of an abnormal thickened dysgenetic dartos fascia bands tethering the corporal bodies proximally and recommended that these bands should be excised for complete correction of such condition.

Another proof of the importance of dartos excision in the case of the concealed penis was concluded by Widi Atmoko et al. [7] in their comparative study in 2018. They compared the histopathology of dartos specimens collected from pediatric patients having hypospadias and buried penis with a control group of normal children and detected an abnormal inelastic dartos fascia in patients with these anomalies. However, this layer has not had the characteristics of fibrous tissue, and, concluded that this abnormal layer should be excised during the repair of both conditions.

Phallopexy was done in our study without excision of dartos fascia in patients of group A and after complete dissection and excision of dartos fascia in patients of group C. This technique was studied by Emrah Aydin et al. [8] for surgical correction of concealed patients with good follow up results and minimal post-operative complications. Phallopexy in the current study was done at three and nine o'clock points in comparison to five and seven O'clock points in the Emrah Aydin et al. [8] study. Applying the stitches at three and nine o'clock points aimed at complete protection of the urethra by being away from it.

Another description of the importance of phallopexy was concluded by Gary et al. [9]. They explained that by the fact that the inadequate attachment of the penile dermal layer to the underlying Buck's fascia allows the corporeal bodies to telescope proximally inside the pubis and scrotum resulting into the buried penis and recommended the fixation of the penile dermis to Buck's fascia at the penoscrotal junction to avoid this telescoping -taking care of the urethra- with good postoperative results.

Penile fixation (phallopexy) after complete dissection and excision of dartos fascia was done in this study with good follow-up results. Joseph [10] described the same good outcome after this technique in his study.

No available study in the literature compared dartos excision alone, phallopexy alone, and the combination of both techniques which was the aim of the current study. The age of the studied patients was slightly lower than that of the patients who were included in the Valioulis et al. [11] study in which the age ranged between three and 12 years with a mean of 54 months. The difference in the median age among the studied groups was significantly different being higher in those patients who underwent dartos excision alone and lowest in those who underwent phallopexy alone.

Follow-up of the studied patients in the current study revealed that most of the studied patients developed post-operative penile edema and only one patient developed skin infection; these complications did not vary significantly among the studied groups and responded well and rapidly to conservative treatment. This observation was similar to the findings of Kassem et al. [1] in their study who concluded that early treatment of buried penis by phallopexy is safe and associated with minor post-operative complications which were also improved rapidly by conservative measures.

None of the studied patients developed post-operative penile skin necrosis as meticulous dissection with great attention to preserving skin vascularity was done. On the other hand; seven patients (4.7%) developed penile skin congestion with gradual and spontaneous improvement. These observations were similar to the findings of Kassem et al. in their study who observed minimal post-operative complications associated with early management of buried penis [1].

Fifteen patients (10%) developed post-operative penile re-retraction with redundant skin over the glans penis. The best results with the lowest incidence of re-retraction were observed in patients who were treated by phallopexy after complete dissection and excision of dartos fascia. However, the highest incidence of this outcome was observed in those who were treated only by dartos excision. Those patients were managed by educating the parents to do regular backward retraction of the skin and meticulous cleaning.

The incidence of post-operative penile re-retraction in our study was higher than that observed by Cheng et al. [12] in their study who found only one patient with penile retraction; their study was conducted on 65 patients (1.5%); this may be attributed to the different approaches in our study with a high re-retraction rate in those patients who were treated by dartos excision only; this procedure was not studied widely in the literature.

Other surgeons believed in the importance of the dartos layer and avoided its excision in the treatment of buried penis and innovated a new technique of doing dorsal dartos flap fixed at both sides of the penile base with satisfactory results as compared to fascia fixation [13].

## Conclusions

This study suggests the addition of dartos excision to phallopexy in the treatment of buried penis as this combination has a lower rate of postoperative penile re-retraction -which is the main postoperative follow-up point- than doing either technique alone. However; further study with sooner follow-up periods at 1st, 3rd, 5th and 7th postoperative days points to document the regression of penile edema. Also, a longer period of follow-up should be carried out in further studies to detect any delayed re-retraction and whether using an absorbable suture is sufficient or should be replaced with a non-absorbable one. Also, a further study on a larger number of patients is recommended to add to the power of the study.

## Data Availability

The data and material of our manuscript are available. "The dataset supporting the conclusions of this article is available in the filing system of our institute. Please contact the corresponding author Ahmed Elrouby if any of this data was requested.
